# Supplementary figures and images for: Potential role of RhoA GTPase regulation in type interferon signaling in systemic lupus erythematosus
Source: Arthritis Res Ther. 2024 Jan 20;26:31. doi: 10.1186/s13075-024-03263-3 (PMC10799493; doi:10.1186/s13075-024-03263-3)

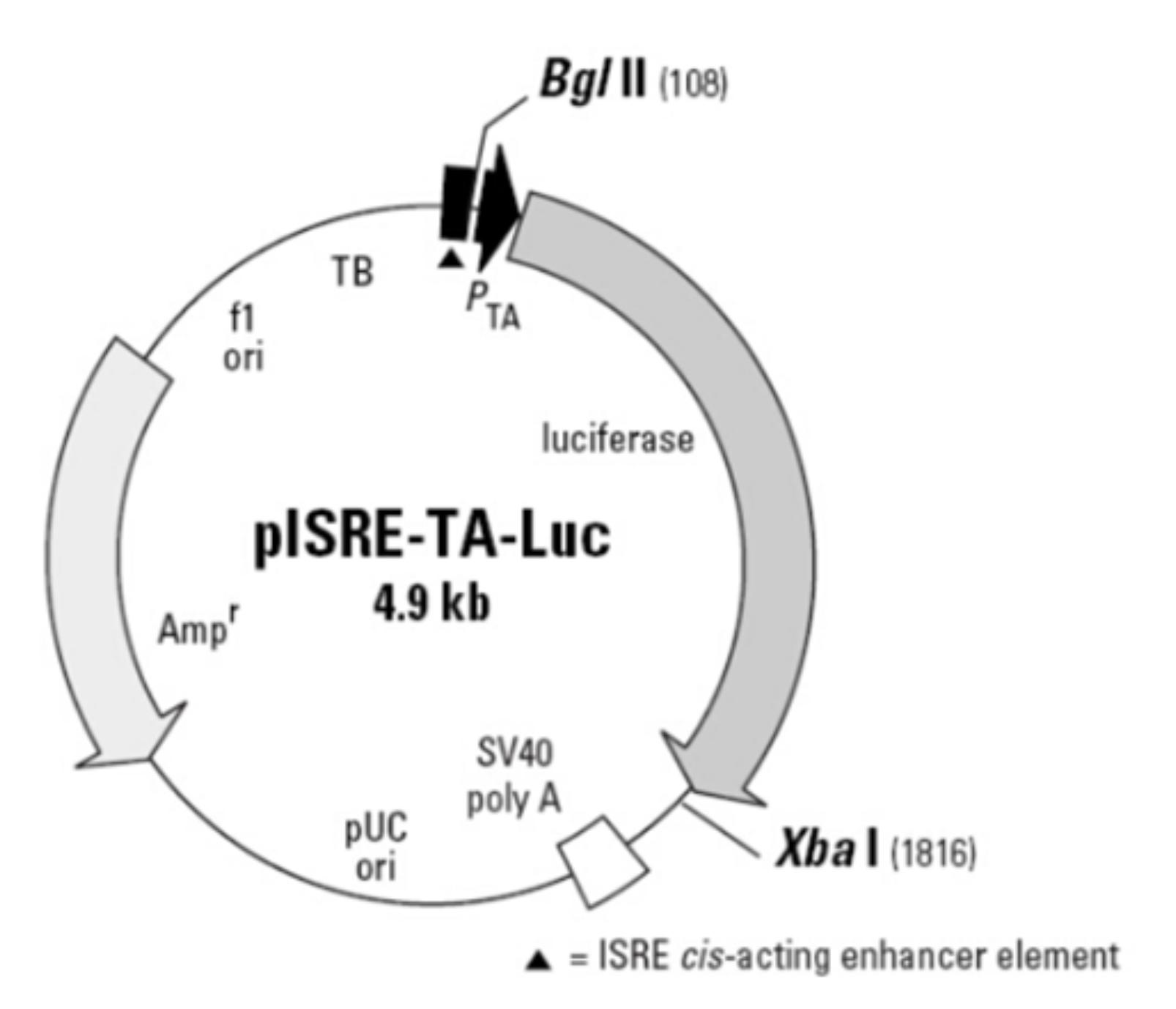

Supplement: Supplementary file 1 — Additional file 1: Supplementary Fig. 1. Plasmid construct. A schematic figure of the plasmid construct is pISRE-TA-Luc for monitoring the induction of the STAT1 and STAT2 components of JAK/STAT-mediated signal transduction pathways. [file 13075_2024_3263_MOESM1_ESM.jpg]

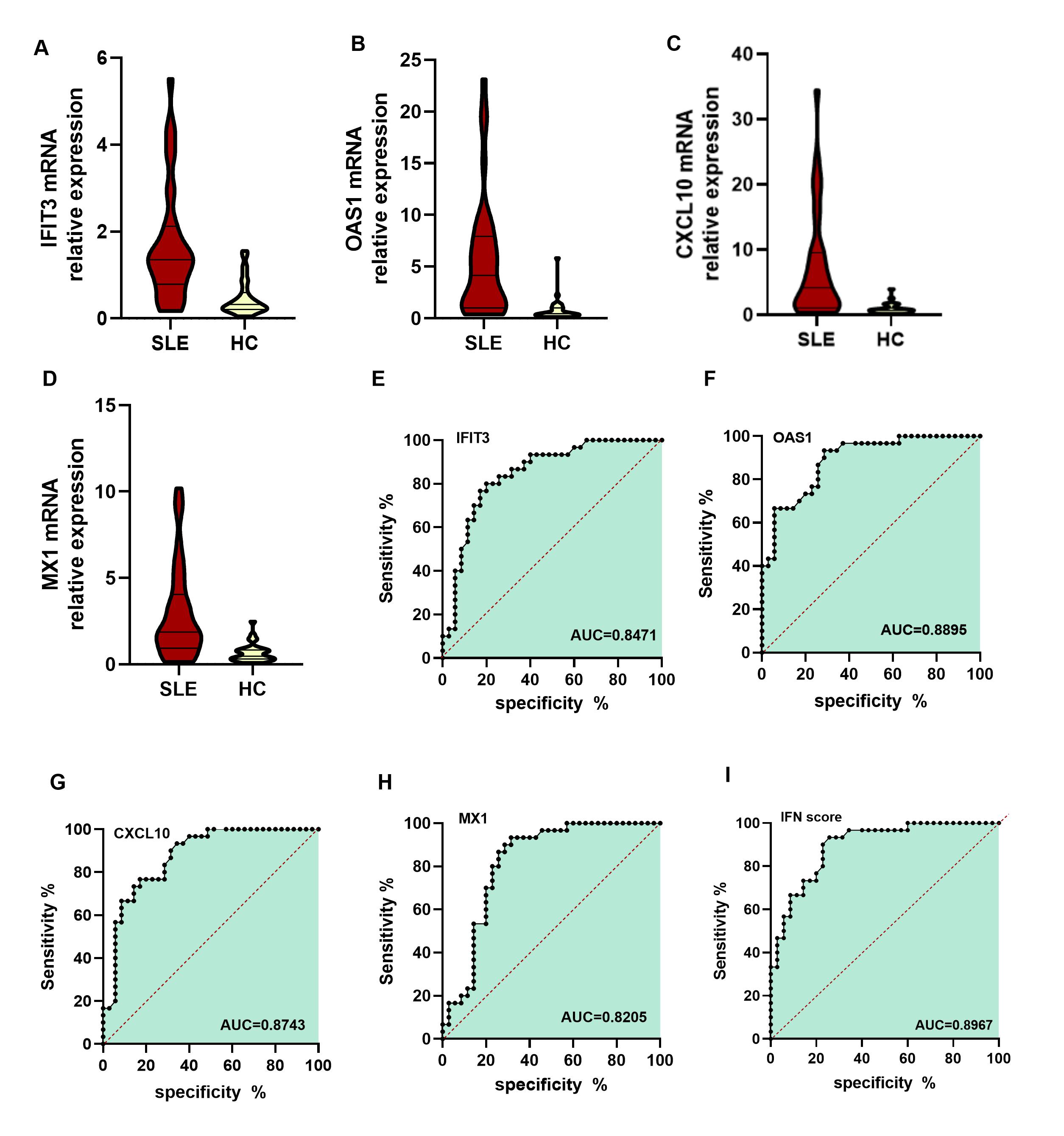

Supplement: Supplementary file 2 — Additional file 2: Supplementary Fig. 2. Increased type I IFN-inducible gene expression in SLE patients. Increased type I IFN-inducible gene (MX1, OAS1, IFIT3 and CXCL10) expression in SLE patients compared to the healthy control group. Area under the receiver operating characteristic curves (AUC) for detection of SLE by references to the expression levels of MX1, OAS1, IFIT3, CXCL10 and the IFN score. Statistical significance was determined using Student’s ttest; the p-values are all less than 0.001. [file 13075_2024_3263_MOESM2_ESM.jpg]

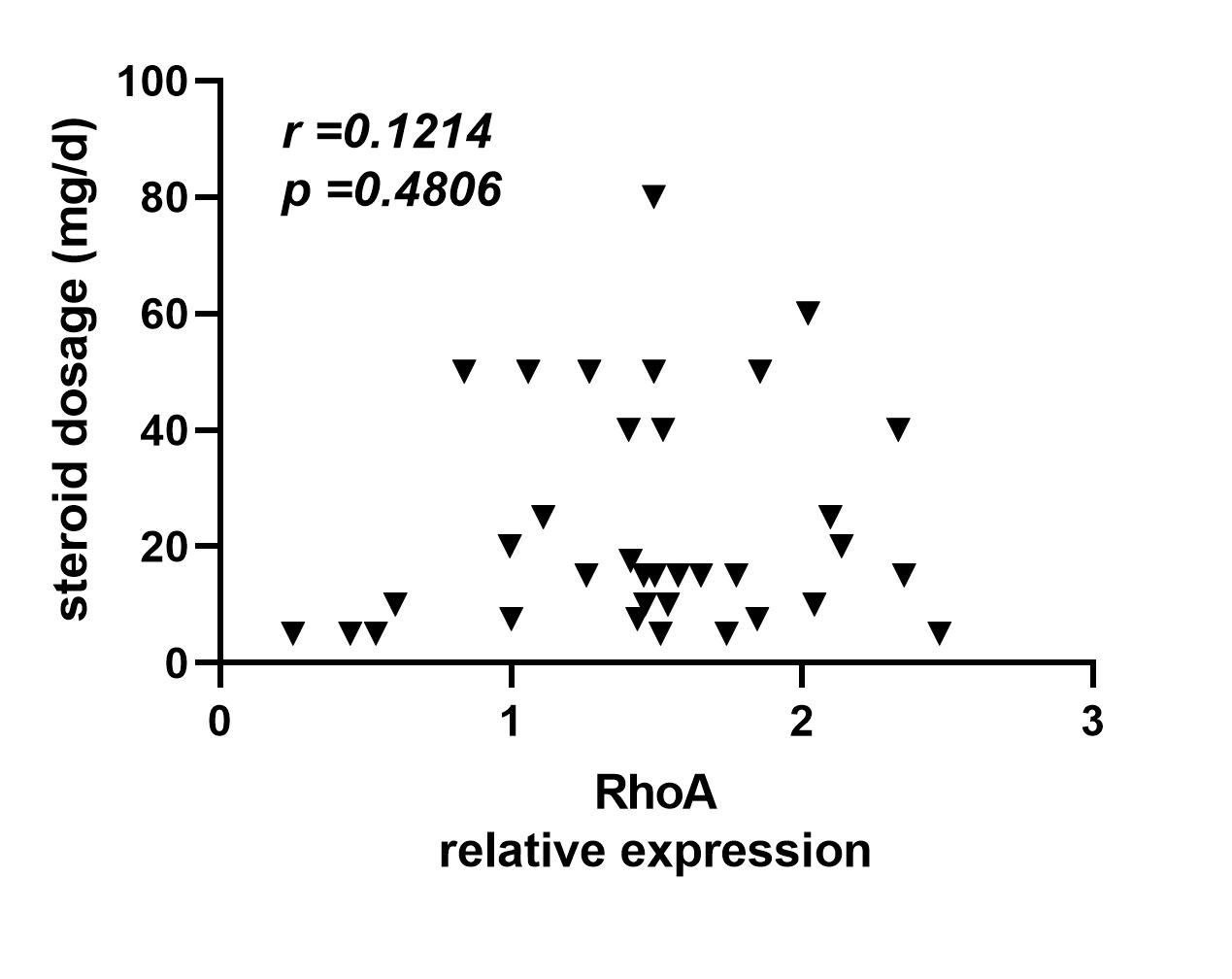

Supplement: Supplementary file 3 — Additional file 3: Supplementary Fig. 3. Correlation between RhoA expression levels and steroid dosage in SLE patients. Each symbol represents an individual patient sample. The dosage of steroids presented refers to prednisone or its equivalent. [file 13075_2024_3263_MOESM3_ESM.jpg]

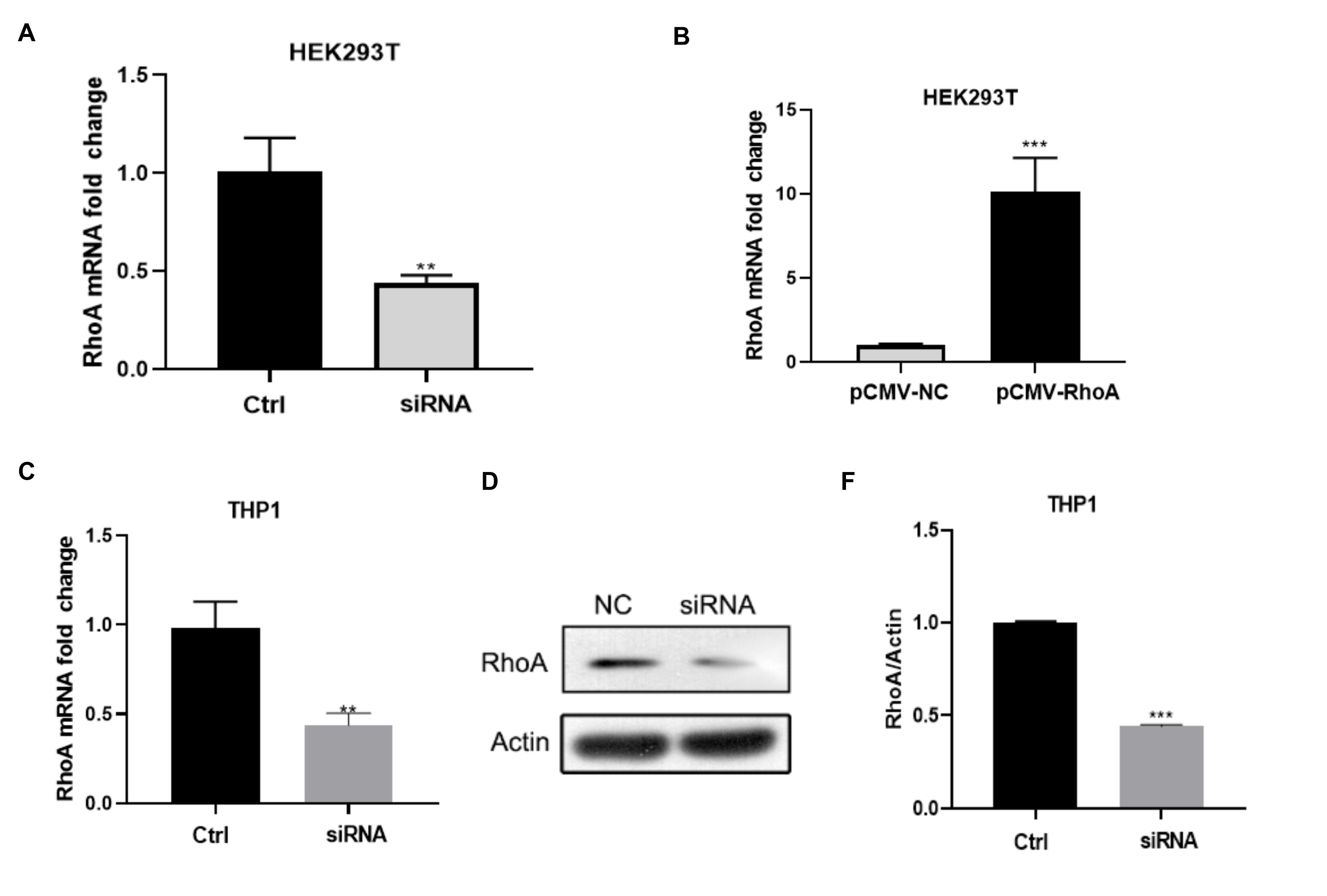

Supplement: Supplementary file 4 — Additional file 4: Supplementary Fig. 4. The expression changes of RhoA after transfection. Quantitative PCR analysis of RhoA expression in HEK-293T 24 hours post-transfection of siRNA targeting RhoA mRNA at 200 nM (A) or a RhoA expression plasmid vector at 4 ug/mL (B), along with their respective controls (a negative control siRNA or pCMV-NC). Quantitative PCR (C) and immunoblot (D) analysis carried out to evaluate the expression changes of RhoA in THP1 cells after transfection with RhoA siRNA or control siRNA directed against RhoA. (E) The histograms represent the ratios of RhoA protein to actin. The immunoblot results of cells transfected with a negative control (Ctrl) were assigned a value of 1. The results are presented as mean±SEM. Statistical significance was determined using Student’s t-test, with **p < 0.01 and ***p < 0.001 denoting significance. [file 13075_2024_3263_MOESM4_ESM.jpg]

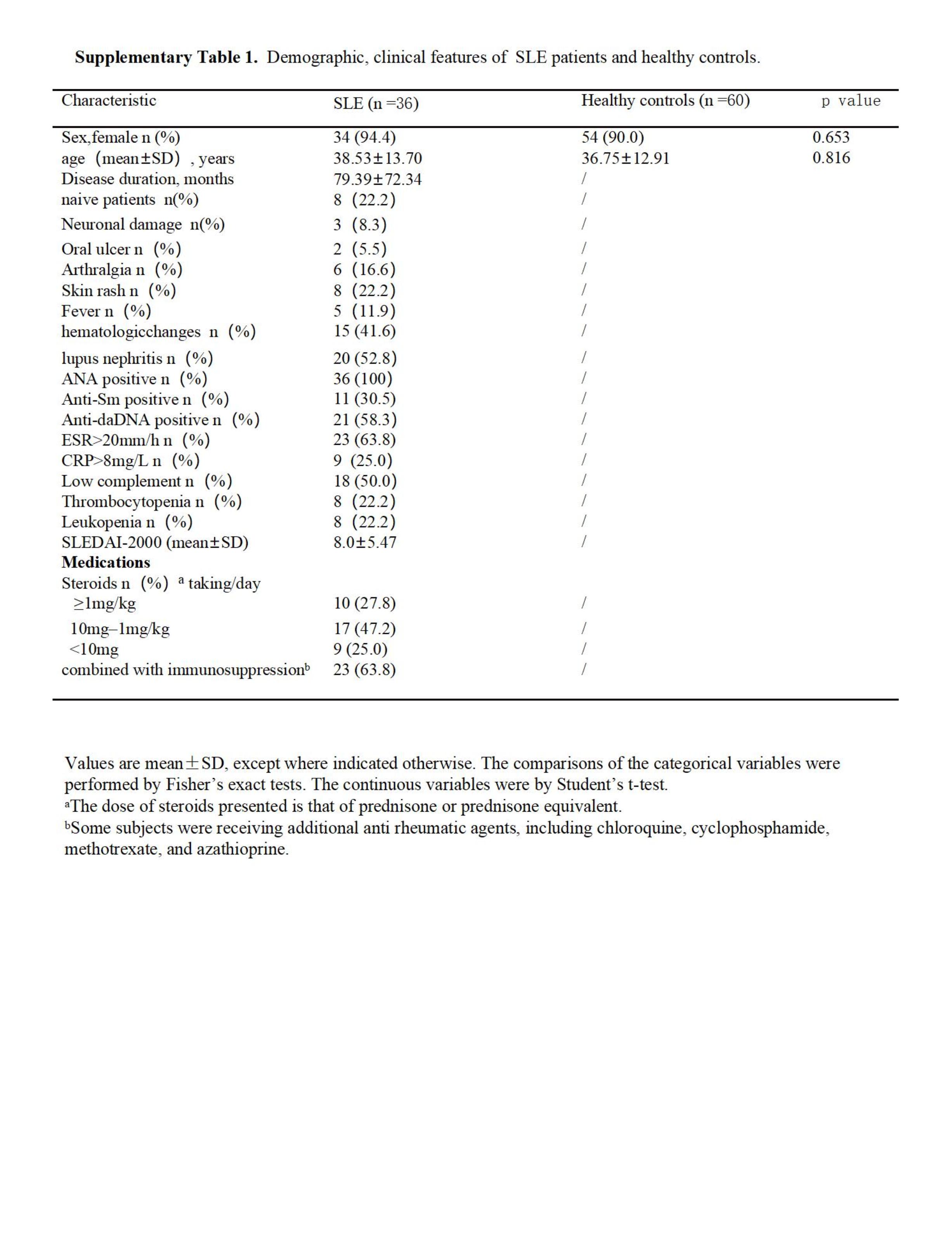

Supplement: Supplementary file 5 — Additional file 5: Supplementary Table 1. Demographic, clinical features of SLE patients and healthy controls. Supplementary Table 2. Demographic and clinical features of SLE patients for the PBMC studies. [file 13075_2024_3263_MOESM5_ESM.zip › Supplementary Table 1.jpg]

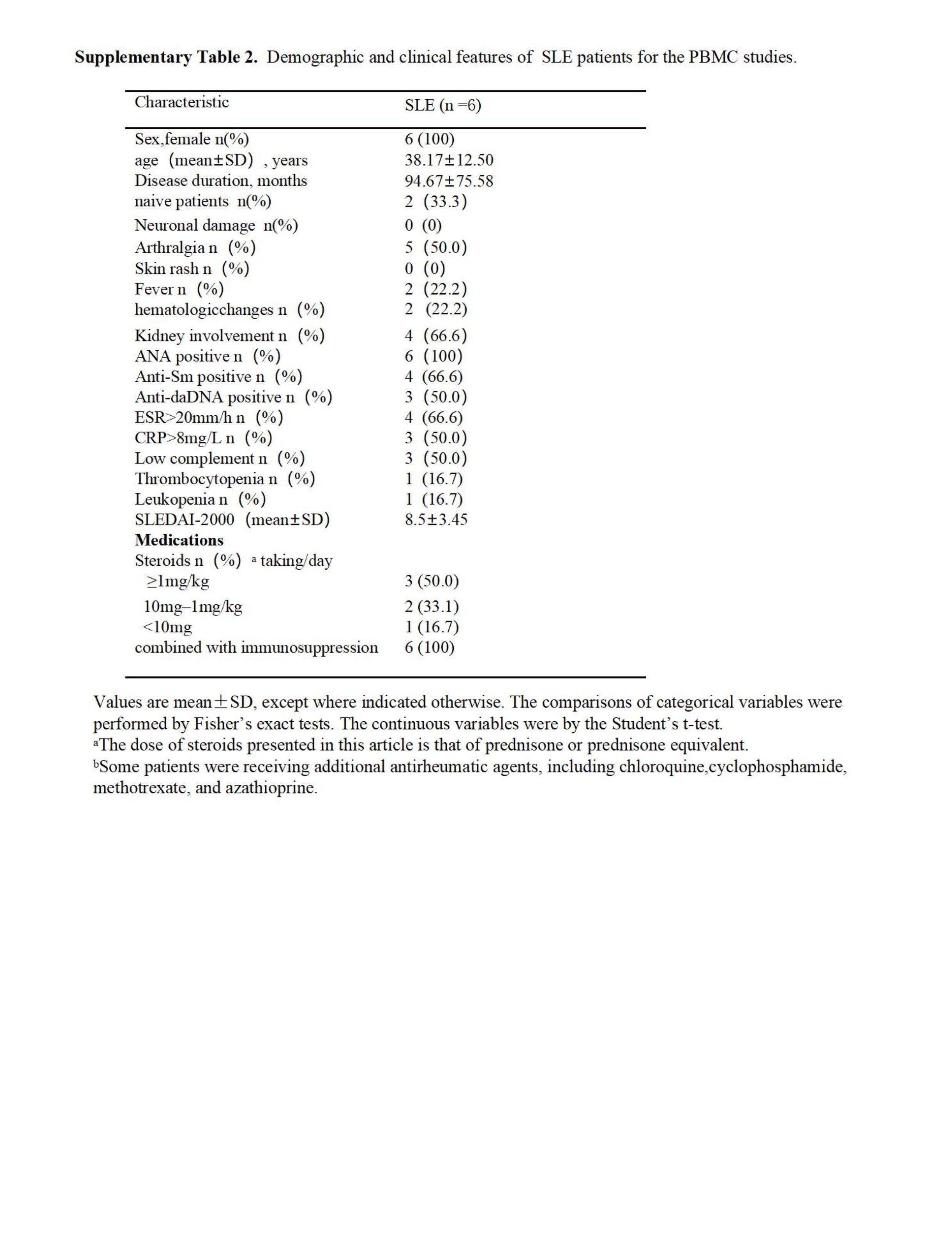

Supplement: Supplementary file 5 — Additional file 5: Supplementary Table 1. Demographic, clinical features of SLE patients and healthy controls. Supplementary Table 2. Demographic and clinical features of SLE patients for the PBMC studies. [file 13075_2024_3263_MOESM5_ESM.zip › Supplementary Table 2.jpg]
